# Angle-Polarization Estimation for Coherent Sources with Linear Tripole Sensor Arrays

**DOI:** 10.3390/s16020248

**Published:** 2016-02-19

**Authors:** Kun Wang, Jin He, Ting Shu, Zhong Liu

**Affiliations:** 1 Department of Electronic Engineering, Nanjing University of Science and Technology, Nanjing 210094, China; wk_1024@163.com (K.W.); eezliu@mail.njust.edu.cn (Z.L.); 2 Shanghai Key Laboratory of Intelligent Sensing and Recognition, Department of Electronic Engineering, Shanghai Jiaotong University, Shanghai 200240, China; tingshu@sjtu.edu.cn

**Keywords:** parallel factor, tripole sensor, angle estimation, polarization estimation

## Abstract

We propose a parallel factor (PARAFAC) analysis-based angle and polarization estimation algorithm for multiple coherent sources using a uniformly-spaced linear tripole sensor array. By forming a PARAFAC model using the spatial signature of the tripole array, the new algorithm requires neither spatial smoothing nor vector-field smoothing to decorrelate the signal coherency. We also establish that the angle-polarization parameters of *K* coherent signals can be uniquely determined by PARAFAC analysis, as long as the number of tripoles L≥2K−1. In addition, the proposed algorithm can offer enhanced angle and polarization estimation accuracy by extending the interspacing of the tripoles beyond a half wavelength.

## 1. Introduction

Estimation of azimuth-elevation angles of multiple electromagnetic signals using the sensor array technique is an important problem encountered in many areas involving radar, seismology and wireless communication. Many existing direction-finding algorithms have been developed using an array of scalar sensors, each of which measures the scalar quantities of the electromagnetic field induced at the sensor [1]. Recently, it has been found that exploiting an array of “polarization-sensitive sensors” can provide measurements of more than one scalar quantity of the electromagnetic field. The electromagnetic vector sensors, crossed-dipoles and tripoles are widely-used polarization-sensitive sensors. An electromagnetic vector sensor can simultaneously obtain the measurements of all six components of the electromagnetic field, whereas a crossed-dipole or a tripole can measure two or three components of the entire electromagnetic field. During the past few decades, many subspace-based techniques have been adapted to polarization-sensitive sensors to estimate 2D angles of the narrowband electromagnetic sources. For example, [2,3,4,5,6,7,8,9,10] investigate the direction-finding methods using electromagnetic vector sensor arrays; [11,12,13,14] investigate the direction-finding methods using crossed-dipoles; and [15,16,17,18,19] investigate the direction-finding methods using tripoles. Since the angle and polarization of the electromagnetic signal are closely related, these methods can offer improved angle estimation performance compared to conventional scalar-sensor array-based methods.

The aforementioned methods are based on the assumption that the impinging source signals are incoherent, with the full rank signal correlation matrix. However, this assumption may be violated in multipath environments [20]. When coherent sources are present, the signal covariance matrix will drop rank, and hence, the performance of the above methods could deteriorate seriously. To cope with coherent signal direction finding, the spatial smoothing technique [21] is often used. In spatial smoothing, sensors are divided into several (possibly overlapping) spatial-shifted groups. The data correlation matrices of these groups are averaged to recover the rank of the signal covariance matrix. A major shortcoming of the spatial smoothing processing is that it reduces the effective array size and, consequently, degrades the angular resolution and estimation performance. For polarization-sensitive sensor arrays, a more sophisticated decoherency method is to perform smoothing by averaging the data correlation matrices corresponding to each electric/magnetic component [3,5]. However, these methods require a planar array structure and/or two-dimensional nonlinear searching to estimate azimuth-elevation directions. Another decoherency method is the so-called subarray averaging [22]. With subarray averaging, the subspace-based method can be applied to estimate the vector sensor array manifolds. However, this method requires additional computations to pair the estimated vector sensor array components.

In this paper, we propose a new two-dimensional angle and polarization estimation algorithm for full correlated sources by employing uniformly-spaced linear tripole arrays. The proposed algorithm formulates a parallel factor (PARAFAC) model by using the spatial signature of the tripole array to extract tripole array manifolds by PARAFAC analysis, without requiring one to perform spatial smoothing or vector-field smoothing to decorrelate the signal coherency. We also establish that the azimuth-elevation directions and polarizations of *K* coherent signals can be uniquely determined by PARAFAC analysis, as long as the number of tripoles L≥2K−1. The proposed algorithm can provide increased parameter estimation precision by extending the interspacing of the tripoles beyond a half wavelength.

## 2. Mathematical Data Model

We consider an *L*-element uniformly-linear tripole array, receiving *K* narrowband completely-polarized planer electromagnetic signals. The *k*-th source signal is parameterized by angle and polarization parameters $\{\theta_{k},\phi_{k},$ $\gamma_{k},$ $\eta_{k}\}$. Each tripole measures the three electric components at a point. The normalized tripole steering vector of the *k*-th signal can be expressed as:

(1) $$\begin{array}{c} \begin{array}{cc} e_{k}= & \left(\sin\gamma_{k}\cos\theta_{k}\cos\phi_{k}e^{j\eta_{k}}-\cos\gamma_{k}\sin\phi_{k}\right){\vec{v}}_{x}\\ & +\left(\sin\gamma_{k}\cos\theta_{k}\sin\phi_{k}e^{j\eta_{k}}+\cos\gamma_{k}\cos\phi_{k}\right){\vec{v}}_{y}\\ & -\sin\gamma_{k}\sin\theta_{k}e^{j\eta_{k}}{\vec{v}}_{z} \end{array} \end{array}$$

where $0\leq \theta_{k}<\pi$, $0\leq \phi_{k}<2\pi$, respectively, represent the elevation and azimuth angles of the *k*-th signal and $0\leq \gamma_{k}<\pi /2$, $-\pi \leq \eta_{k}<\pi$, are the corresponding polarization parameters. ${\vec{v}}_{x}$, ${\vec{v}}_{y}$, ${\vec{v}}_{z}$ are respectively the unit vector along the three Cartesian coordinates. Expressing Equation (1) in matrix form, we have the following 3×1 vector, which represents the tripole’s steering vector for the *k*-th signal:

(2) $$\begin{array}{c} c_{k}=\left[\begin{array}{c} c_{1,k}\\ c_{2,k}\\ c_{3,k} \end{array}\right]=\left[\begin{array}{c} \sin\gamma_{k}\cos\theta_{k}\cos\phi_{k}e^{j\eta_{k}}-\cos\gamma_{k}\sin\phi_{k}\\ \sin\gamma_{k}\cos\theta_{k}\sin\phi_{k}e^{j\eta_{k}}+\cos\gamma_{k}\cos\phi_{k}\\ -\sin\gamma_{k}\sin\theta_{k}e^{j\eta_{k}} \end{array}\right] \end{array}$$

Note that, unlike the steering vector of planar arrays, the tripole’s steering vector does not contain angle-dependent phase progression. This property is crucial in improving the estimation performance by extending the tripole interspacing beyond a half wavelength.

The spatial response related to the *k*-th signal and the *ℓ*-th tripole is:

(3) $$\begin{array}{c} q_{\ell }\left(\theta_{k},\phi_{k}\right)=e^{j2\pi \frac{x_{\ell }u_{k}}{\lambda }}e^{j2\pi \frac{y_{\ell }v_{k}}{\lambda }}=e^{j2\pi \frac{x_{\ell }u_{k}+y_{\ell }v_{k}}{\lambda }} \end{array}$$

where $u_{k}=\sin\theta_{k}\cos\phi_{k}$, $v_{k}=\sin\theta_{k}\sin\phi_{k}$ represent the direction cosines along the *x*-axis and *y*-axis and $\left(x_{\ell },y_{\ell }\right)$ is the location of the *ℓ*-th tripole. Then, the entire tripole array has the following 3L×1 manifold:

(4) $$\begin{array}{ccc} a(\theta_{k},\phi_{k},\gamma_{k},\eta_{k}) & = & \left[\begin{array}{c} a_{1}\left(\theta_{k},\phi_{k},\gamma_{k},\eta_{k}\right)\\ \vdots \\ a_{L}\left(\theta_{k},\phi_{k},\gamma_{k},\eta_{k}\right) \end{array}\right]=\left[\begin{array}{c} q_{1}\left(\theta_{k},\phi_{k}\right)\\ \vdots \\ q_{L}\left(\theta_{k},\phi_{k}\right) \end{array}\right]⊗c_{k}=q\left(\theta_{k},\phi_{k}\right)⊗c_{k} \end{array}$$

The 3L×1 output vector of the entire tripole array, measured at time *n*, can be expressed as:

(5) $$\begin{array}{c} z\left(n\right)=\sum_{k=1}^{K}a\left(\theta_{k},\phi_{k},\gamma_{k},\eta_{k}\right)s_{k}\left(n\right)+v\left(n\right)=As\left(n\right)+v\left(n\right) \end{array}$$

where $s\left(n\right)\overset{\mathrm{def}}{=}{\left[s_{1}\left(n\right),\cdots ,s_{K}\left(n\right)\right]}^{T}$, with $s_{k}\left(n\right)$ the waveform of the *k*-th signal; $A\overset{\mathrm{def}}{=}$ $[a(\theta_{1},\phi_{1},$$\gamma_{1},\eta_{1}),$⋯,$a(\theta_{K},\phi_{K},$$\gamma_{K},\eta_{K})]$; $v\left(n\right)\overset{\mathrm{def}}{=}{\left[v_{1}\left(n\right),\cdots ,v_{3L}\left(n\right)\right]}^{T}$ represents the 3L×1 additive noise. The noise is assumed to be independent to all signals. Further, the signals are considered as fully coherent, so that they can be expressed as complex multiples of the signal $s_{1}\left(n\right)$, *i.e*.,

(6) $$\begin{array}{c} s_{k}\left(n\right)=\beta_{k}s_{1}\left(n\right) \end{array}$$

where $\beta_{k}$ is the complex coefficient with $\beta_{k}\neq 0$ and $\beta_{1}=1$.

The target of the paper is to estimate the angle and polarization parameters $\{\theta_{k},\phi_{k},$$\gamma_{k},\eta_{k},$k=1,⋯,K} from the *N* data samples z(1),⋯,z(N) measured at the time instants {n=1,⋯,N}.

## 3. Proposed Solution

The vector z(n) in Equation (5) can be reexpressed as:

(7) $$\begin{array}{c} z\left(n\right)=A\beta s_{1}\left(n\right)+v\left(n\right)=bs_{1}\left(n\right)+v\left(n\right) \end{array}$$

where:

(8) $$\begin{array}{c} \beta ={\left[\beta_{1},\beta_{2},\cdots ,\beta_{K}\right]}^{T} \end{array}$$

(9) $$\begin{array}{c} b=\sum_{k=1}^{K}\beta_{k}a\left(\theta_{k},\phi_{k},\gamma_{k},\eta_{k}\right) \end{array}$$

is the spatial signature of the tripole array, which contains sufficient information on signal direction $\left(\theta_{k},\phi_{k}\right)$ and polarization $\left(\gamma_{k},\eta_{k}\right)$. The proposed algorithm is based on the formulation of a PARAFAC model from the vector b.

### 3.1. PARAFAC Model Formulation

The PARAFAC model is a useful data analysis tool, originating from psychometrics [23,24] in 1970. In recent years, it has been found in various applications, such as in sensor array signal processing [1], communications [25] and biology [26].

To formulate the PARAFAC model, we reshape the 3L×1 vector b to a 3×L matrix as:

(10) $$\begin{array}{c} Z=\underset{C}{\underset{︸}{\left[c_{1},\cdots ,c_{K}\right]}}\underset{Q}{\underset{︸}{{\left[\beta_{1}q\left(\theta_{1},\phi_{1}\right),\cdots ,\beta_{K}q\left(\theta_{K},\phi_{K}\right)\right]}^{T}}}={\mathrm{CQ}}^{T} \end{array}$$

From Equation (10), with a total of *L* tripoles, we can form P(P≥2) different spatial-shifted datasets, where each associates with (L−P+1) tripoles. The *p*-th spatial-shifted dataset has the form:

(11) $$\begin{array}{c} Z_{p}=C\Phi_{p}Q_{1}^{T},\;p=1,\cdots ,P \end{array}$$

where:

(12) $$\begin{array}{c} \Phi_{p}=\mathrm{diag}\left[e^{-j\omega_{1}\left(p-1\right)},\cdots ,e^{-j\omega_{K}\left(p-1\right)}\right] \end{array}$$

with $\omega_{k}=\frac{2\pi }{\lambda }\left(\Delta_{x}u_{k}+\Delta_{y}v_{k}\right)$ a diagonal matrix and $Q_{1}$ the first (L−P+1) rows of Q. Then, for p=1,⋯,P, we will have *P* different datasets $\left\{Z_{1},\cdots ,Z_{P}\right\}$. Since the matrices $\Phi_{p}$ are different from one set to another, these *P* datasets differ from each other. Next, stacking these *P* matrices, we can form a three-way array $\underline{Z}$, of which the (i,j,p)-th element is written as:

(13) $$\begin{array}{c} z_{i,j,p}={\left[\underline{Z}\right]}_{i,j,p}=\sum_{k=1}^{K}c_{i,k}\varphi_{p,k}\alpha_{j,k} \end{array}$$

where i=1,⋯,3, j=1,⋯,N−P+1, p=1,⋯,P, $c_{i,k}$ and $\alpha_{j,k}$, respectively, denote the (i,k)-th and the (j,k)-th entries of C and $Q_{1}$, and $\varphi_{p,k}$ represents the (p,k)-th element of the matrix **Ψ** defined as:

(14) $$\begin{array}{c} \Psi =\left[\begin{array}{cccc} 1 & 1 & \cdots & 1\\ e^{-j\omega_{1}} & e^{-j\omega_{2}} & \cdots & e^{-j\omega_{K}}\\ \vdots & \vdots & \cdots & \vdots \\ e^{-j\omega_{1}\left(P-1\right)} & e^{-j\omega_{2}\left(P-1\right)} & \cdots & e^{-j\omega_{K}\left(P-1\right)} \end{array}\right] \end{array}$$

Apparently, the matrices $\Phi_{p}$ and **Ψ** have the following relationship:

(15) $$\begin{array}{c} \Phi_{p}=D_{p}\left\{\Psi \right\},\;\forall p=1,\cdots ,P \end{array}$$

where $D_{p}\left\{\cdot \right\}$ denotes the operator, which produces a diagonal matrix by using the elements in the *p*-th row of the matrix in brackets.

In Equation (13), $z_{i,j,p}$ is expressed as a sum of *K* rank one triple products. Equation (13) also denotes a unique low-rank decomposition of $\underline{Z}$, provided that certain conditions are satisfied. Therefore, the problem under consideration is identical to that of the low-rank decomposition of the three way array (TWA) $\underline{Z}$. The latter can be solved by PARAFAC fitting.

### 3.2. PARAFAC Model Identifiability

In this subsection, we will discuss the identifiability condition for unique low-rank decomposition of $\underline{Z}$. The discussion is based on the definition of the Kruskal rank of a matrix [27].

*Definition* : The Kruskal rank (or *k*-rank) of a matrix A is $k_{A}$, if and only if every $k_{A}$ column of A is linearly independent and either A has $k_{A}$ columns or A contains a set of $k_{A}+1$ linearly-dependent columns. Note that the Kruskal rank is always not greater than the conventional matrix rank. If A is of full column rank, then it is of full *k*-rank, as well.

To establish the identifiability, by using the relationship in Equation (15), we can rewrite the three-way array $\underline{Z}$ in a compact way as:

(16) $$\begin{array}{c} \bar{Z}=\left[\begin{array}{c} Z_{1}\\ Z_{2}\\ \vdots \\ Z_{P} \end{array}\right]=\left[\begin{array}{c} CD_{1}\left(\Psi \right)\\ CD_{2}\left(\Psi \right)\\ \vdots \\ CD_{P}\left(\Psi \right) \end{array}\right]Q_{1}^{T}=\left(\Psi ⊙C\right)Q_{1}^{T} \end{array}$$

where ⊙ is the Khatri–Rao product operator. The identifiability results are based on the the following theorem.

**Theorem 1.** *For an I×J×K TWA $\underline{X}$ with a typical element $x_{i,j,k}=\sum_{f=1}^{F}a_{i,f}b_{j,f}c_{k,f}$, i=1,⋯,I,j=1,⋯,J,k=1,⋯,K, where $a_{i,f}$, $b_{j,f}$ and $c_{k,f}$ respectively stand for the (i,f)-th, (j,f)-th and (k,f)-th elements of the I×F, J×F and K×F matrices A, B and C. If for F>1,*

(17) $$\begin{array}{c} k_{A}+k_{B}+k_{C}\geq 2F+2 \end{array}$$

*then A, B and C are unique up to unresolvable permutation and scaling ambiguities [25]*. 

On the basis of Theorem 1, we provide the sufficient conditions for the number of tripoles to guarantee the models in Equation (16) to be identifiable.

**Theorem 2.** *The sufficient identifiable conditions for the TWAs Equation (16) are L≥2K−1*.

**Proof:** We know that for a tripole, every two tripole response vectors with distinct angle and polarization parameters are linearly independent. This means that the inequality $k_{C}\geq 2$ holds. Next, for a uniformly-linear array, matrices **Ψ** and $Q_{1}$ are of Vandermonde structures, and hence, they are of full column rank. Therefore, the following two equalities hold:

(18) $$\begin{array}{ccc} k_{\Psi } & = & \mathrm{rank}\{\Psi \}=\min(K,P) \end{array}$$

(19) $$\begin{array}{ccc} k_{Q_{1}} & = & \mathrm{rank}\left\{Q_{1}\right\}=\min\left(K,L-P+1\right) \end{array}$$

Substituting Equations (18) and (19) into Equation (17), we have the identifiable condition:

(20) $$\begin{array}{c} k_{C}+\min\left(K,P\right)+\min\left(K,L-P+1\right)\geq 2K+2 \end{array}$$

We discuss the following four cases:

1. P<K, L−P+1<K. This implies that L<2K−1. In this case, in order to satisfy Equation (20), we have L≥2K−1. This is contradictory with L<2K−1. Therefore, the TWA Equation (16) is not identifiable for this case.
2. P≥K, L−P+1<K. In this case, in order to satisfy Equation (20), we have L−P+1≥K. This is contradictory with L−P+1<K. Therefore, the TWA Equation (16) is not identifiable for this case.
3. P<K, L−P+1≥K. In this case, in order to satisfy Equation (20), we have P≥K. This is contradictory with P<K. Therefore, the TWA Equation (16) is not identifiable for this case.
4. P≥K, L−P+1≥K. In this case, Equation (20) becomes $k_{C}+K+K\geq 2K+2$. Since $k_{C}\geq 2$ is always satisfied, the TWA Equation (16) is always identifiable for this case. Furthermore, P≥K and L−P+1≥K together lead to L≥2K−1.

With the above discussions, the results in Theorem 2 are established.

### 3.3. PARAFAC Fitting

For the PARAFAC fitting problem, many effective algorithms have been proposed. For the purpose of illustration, the trilinear alternating least square (TALS) algorithm [25] is considered in this paper. Firstly, the unknown parameters are divided into three sets. Secondly, a least squares problem that depends only on one set is optimized. Thirdly, with this least squares solution, the subsequent stages are to solve the least squares problems on the remaining two parameter sets. Finally, perform iterations from set to set, until some convergence criterion is satisfied. The TALS algorithm is guaranteed to be monotonically converged, because all of the optimizations are solved via the least squares criterion.

With the estimation of $\hat{C}$ by PARAFAC fitting, the angles and polarizations of the impinging sources can then be recovered. It should be noted that the estimate $\hat{C}$ is unique, except some unknown scaling and permutation ambiguities. The former can be easily resolved by normalizing each column of $\hat{C}$ with respect to its first element. The latter is unresolvable; however, it is usually extraneous, since the ordering of the estimated parameters is unimportant.

The normalized version of ${\hat{c}}_{k}$ can be expressed as:

(21) $$\begin{array}{c} {\hat{c}}_{k}=ae^{j\omega }c_{k} \end{array}$$

where *a* and *ω* are unknown real-valued amplitude and phase. Then, referring to Equation (21), the the real and imaginary entries of ${\hat{c}}_{k}$ can be used to form the following nonlinear equations:

−cosθkcosϕksinγk+cosγksinϕkcosηk=aRe{[c^k]1e−j∠[c^k]3}−cosθksinϕksinγk−cosγkcosϕkcosηk=aRe{[c^k]2e−j∠[c^k]3}sinθksinγk=aRe{[c^k]3e−j∠[c^k]3}−cosγksinϕksinηk=aIm{[c^k]1e−j∠[c^k]3}cosγkcosϕksinηk=aIm{[c^k]2e−j∠[c^k]3}

Finally, the angle and polarization parameters can be easily obtained by solving the above equations (please refer to [4] for detailed steps).

### 3.4. Estimation of the Spatial Signature of the Tripole Array

From the foregoing analysis, we can infer that with the estimation of the spatial signature of tripole array $\hat{b}$, the angles and polarizations of the signals can be estimated by forming a PARAFAC model from $\hat{b}$ and then solving the formulated PARAFAC decomposition problem. Assuming zero-mean temporally and spatially-uncorrelated noise with variance $\sigma^{2}$, the array covariance matrix is given as:

(22) $$\begin{array}{c} R=E\left\{z\left(n\right)z^{H}\left(n\right)\right\}=\underset{\sigma_{s}^{2}}{\underset{︸}{E\{s_{1}\left(n\right)s_{1}^{*}\left(n\right)\}}}bb^{H}+\sigma^{2}I_{3L} \end{array}$$

Obviously, the rank of noiseless covariance matrix $R-\sigma^{2}I_{3L}$ is clearly equal to one due to the coherency of signals, and its eigenvalue decomposition is given by:

(23) $$\begin{array}{c} R-\sigma^{2}I_{3L}=U\Gamma U^{H}=\lambda_{1}u_{1}u_{1}^{H} \end{array}$$

where $U=[u_{1},\cdots ,u_{3L}]$, $\Gamma =\mathrm{diag}(\lambda_{1},\cdots ,\lambda_{3L})$, $\lambda_{i}$ and $u_{i}$ are eigenvalues and eigenvectors, with $\lambda_{1}>\lambda_{2}=\lambda_{3}=\cdots ,=\lambda_{3L}=0$.

From Equations (22) and (23), we can infer that the vector b can be estimated by the principal eigenvector of $R-\sigma^{2}I_{3L}$. In practical, the noise variance $\sigma^{2}$ can be computed as the average of 3L−1 smallest eigenvalues of R.

### 3.5. Computational Complexity

The major computational complexity of the proposed algorithm is to solve the TALS algorithm. In each iteration, the TALS algorithm includes three LS updates for estimating $Q_{1}$, **Ψ** and C. The computational load for updating $Q_{1}$ is $O[3PK+K^{2}\left(K+6P\right)+3PK\left(N-P+1\right)]$, for updating **Ψ** is $O[3\left(N-P+1\right)K+K^{2}\left(K+6\left(N-P+1\right)\right)+3PK\left(N-P+1\right)]$ and for updating C is $O[P\left(N-P+1\right)K+K^{2}\left(K+2P\left(N-P+1\right)\right)+3PK\left(N-P+1\right)]$. The resulting total computational complexity for each iteration is in the order of $O\{3K^{3}+\left[6\left(N+1\right)+2P\left(N-P+1\right)\right]K^{2}+\left[3\left(N-P+1\right)\left(P+1\right)+7P\left(N-P+1\right)+3P\right]K\}$.

### 3.6. Remarks

It should be mentioned that the application of PARAFAC analysis for angle and/or polarization estimation has already been discussed in [1,6,7,10,19]. However, the underlying assumptions and algorithmic mechanism of the presented work are different from those in the above-mentioned works. For example, [1] formulates the PARAFAC model using a planar array with multiple spatial invariances. The work in [6] links the PARAFAC model with electromagnetic vector sensor arrays and discusses the identification problem for both uncorrelated and coherent signal cases. The work in [7] investigates the regularized PARAFAC analysis for a single electromagnetic vector sensor. The work in [10] forms the PARAFAC model using multiple nested linear arrays. The work in [19] solves the near-field source localization problem using PARAFAC analysis. Moreover, most of the these algorithms assume incoherent signals, so that they cannot be applied to the present problem without performing additional computations to decorrelate the signal coherency.

The presented algorithm obtains azimuth-elevation angle and polarization estimates by performing PARAFAC fitting to a TWA formulated from the spatial signature of the tripole array. The superiorities of the proposed algorithm over other competitive algorithms are summarized as:

1. It requires neither spatial smoothing nor vector-field smoothing to decorrelate the signal coherency.
2. It uses a uniformly-linear array to obtain two-dimensional angle estimation.
3. The estimated azimuth and elevation angles and polarizations are paired automatically without performing any additional pairing computations.

## 4. Simulations

We provide several simulation results to compare the proposed algorithm to the polarization smoothing algorithm [3], the subspace-based method without eigendecomposition (SUMWE) algorithm [22] and the trilinear-based algorithm [6]. A uniform linear array (ULA) with 16 tripoles is used for the proposed algorithm and the trilinear-based one. Note that the polarization smoothing algorithm and the SUMWE algorithm are not suitable for using a linear array to estimate the two-dimensional angles of the signals. We hence use an L-shaped array geometry for these two algorithms instead. For the polarization smoothing algorithm, eight *x*-axis tripoles and eight *y*-axis tripoles are considered. For the SUMWE algorithm, 24 *x*-axis scalar sensors and 24 *y*-axis scalar sensors are used. Hence, the total sensor elements of the four algorithms are identical. Two narrowband Gaussian-distributed signals are assumed to impinge upon the aforementioned arrays. The RMSEs (root mean squared errors) of the parameters are calculated from 500 independent trials.

**Figure 1:**
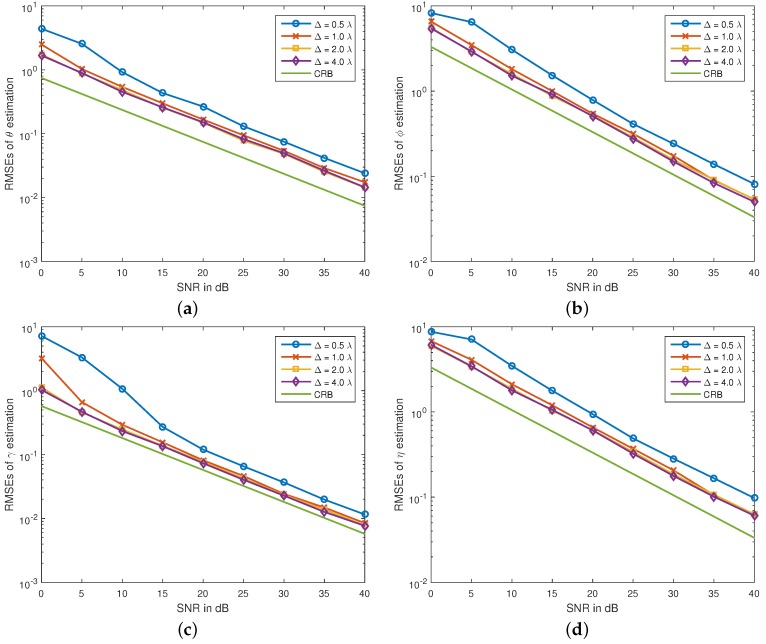
RMSEs of (**a**) $\theta_{1}$, (**b**) $\phi_{1}$, (**c**) $\gamma_{1}$ and (**d**) $\eta_{1}$ estimates *versus* SNRs. The signal parameters are: $\theta_{1}=10{}^{\circ}$, $\phi_{1}=25{}^{\circ}$, $\gamma_{1}=30{}^{\circ}$, $\eta_{1}=-90{}^{\circ}$, $\theta_{2}=20{}^{\circ}$, $\phi_{2}=40{}^{\circ}$, $\gamma_{2}=45{}^{\circ}$ and $\eta_{2}=90{}^{\circ}$, N=200.

Firstly, we plot RMSEs of the proposed algorithm *versus* SNR at different interspacing in Figure 1. The signal parameters are: $\theta_{1}=10{}^{\circ}$, $\phi_{1}=25{}^{\circ}$, $\gamma_{1}=30{}^{\circ}$, $\eta_{1}=-90{}^{\circ}$, $\theta_{2}=20{}^{\circ}$, $\phi_{2}=40{}^{\circ}$, $\gamma_{2}=45{}^{\circ}$ and $\eta_{2}=90{}^{\circ}$. The number of snapshots is N=200. From the figure, we see that the estimation errors of the proposed algorithm decrease with the increasing tripole interspacing. This phenomenon can be explicated intuitively as the extension of the array aperture. For the scalar sensor array, extending the array aperture is known to provide enhanced angle estimation accuracy, but resulting in ambiguous estimation. However, for the tripole array, the proposed algorithm does not suffer estimation ambiguity; even the interspacing of the tripoles exceeds the Nyquist half-wavelength upper limit. This fact is explained as that the new algorithm gives the parameter estimates from the tripole steering vectors, not from the spatial phase factors among the sensors.

**Figure 2:**
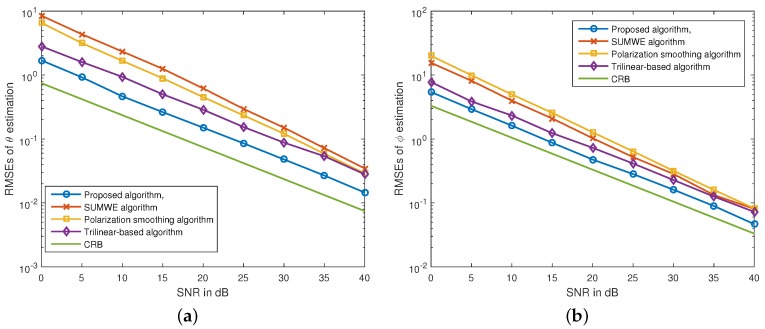
RMSEs of (**a**) $\theta_{1}$ and (**b**) $\phi_{1}$ estimates of the different algorithms *versus* SNRs. The signal parameters are: $\theta_{1}=10{}^{\circ}$, $\phi_{1}=25{}^{\circ}$, $\gamma_{1}=30{}^{\circ}$, $\eta_{1}=-90{}^{\circ}$, $\theta_{2}=20{}^{\circ}$, $\phi_{2}=40{}^{\circ}$, $\gamma_{2}=45{}^{\circ}$ and $\eta_{2}=90{}^{\circ}$, N=200.

Secondly, we show the RMSE results of the presented algorithm, the polarization smoothing algorithm, the SUMWE algorithm and the trilinear-based algorithm as a function of SNR in Figure 2, where the Cramér-Rao bound (CRB) [28] is also shown for comparison. The signal parameters are set the same as those in Figure 1. For the proposed algorithm, we assume the interspacing of tripoles to be four wavelengths. The curves in Figure 2 verify that the proposed algorithm can offer better performance than those of the other three algorithms, in terms of lower estimation RMSEs.

**Figure 3:**
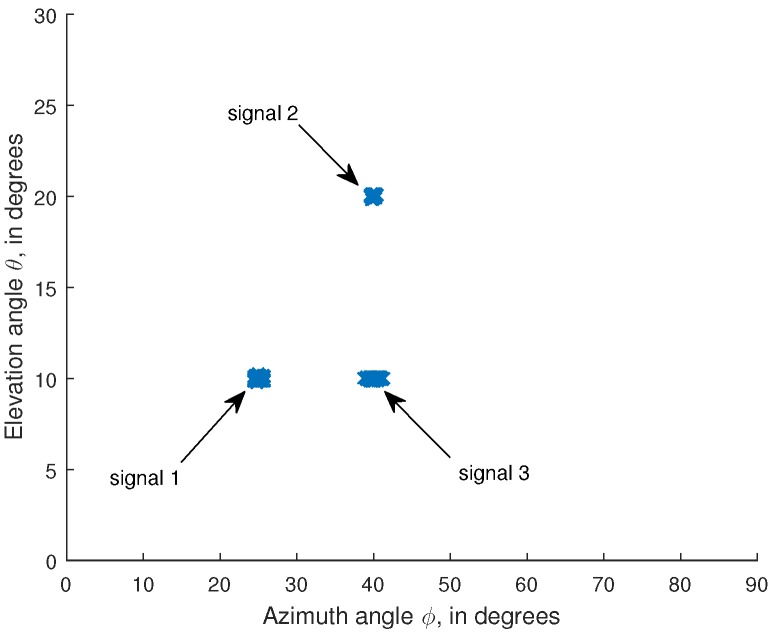
Angle estimation result of the proposed algorithm. The signal parameters are: $\theta_{1}=10{}^{\circ}$, $\phi_{1}=25{}^{\circ}$, $\gamma_{1}=30{}^{\circ}$, $\eta_{1}=-90{}^{\circ}$, $\theta_{2}=20{}^{\circ}$, $\phi_{2}=40{}^{\circ}$, $\gamma_{2}=45{}^{\circ}$, $\eta_{2}=90{}^{\circ}$, $\theta_{3}=10{}^{\circ}$, $\phi_{3}=40{}^{\circ}$, $\gamma_{3}=60{}^{\circ}$, $\eta_{3}=-90{}^{\circ}$. N=200.

Finally, we examine the performance of the presented algorithm for the scenario that some of the signals have the same azimuth angles or elevation angles. In this simulation, we consider that three signals are coming with the angle and polarization parameters: $\theta_{1}=10{}^{\circ}$, $\phi_{1}=25{}^{\circ}$, $\gamma_{1}=30{}^{\circ}$, $\eta_{1}=-90{}^{\circ}$, $\theta_{2}=20{}^{\circ}$, $\phi_{2}=40{}^{\circ}$, $\gamma_{2}=45{}^{\circ}$, $\eta_{2}=90{}^{\circ}$, $\theta_{3}=10{}^{\circ}$, $\phi_{3}=40{}^{\circ}$, $\gamma_{3}=60{}^{\circ}$, $\eta_{3}=-90{}^{\circ}$. The remaining parameters are the same as those used in the first simulation. Figure 3 shows the angle estimation result of the proposed algorithm. It is seen from Figure 3 that the proposed algorithm can still work if some of the signals may have the same azimuth angles or elevation angles.

## 5. Conclusions

We have presented a new algorithm for estimating two-dimensional angles and polarizations for coherent sources using a uniformly-spaced linear tripole array. The PARAFAC model of the spatial signature of the tripole array has been formulated. The resulting estimator for estimating the azimuth and elevation angles and polarization parameters can be estimated by PARAFAC fitting. The new algorithm requires neither spatial smoothing nor vector-field smoothing to decorrelate the signal coherency. The identifiability condition for PARAFAC fitting has been established, as well.
